# The emerging roles of Hedgehog signaling in tumor immune microenvironment

**DOI:** 10.3389/fonc.2023.1171418

**Published:** 2023-05-05

**Authors:** Juan Wang, Baiping Cui, Xiaojie Li, Xinyue Zhao, Taomin Huang, Xiaolei Ding

**Affiliations:** ^1^ Institute of Geriatrics, Affiliated Nantong Hospital of Shanghai University (The Sixth People’s Hospital of Nantong), School of Medicine, Shanghai University, Nantong, China; ^2^ Shanghai Engineering Research Center of Organ Repair, School of Medicine, Shanghai University, Shanghai, China; ^3^ Department of Pharmacy, Eye & ENT Hospital, Fudan University, Shanghai, China

**Keywords:** Hedgehog signaling pathway, tumor immunity, tumor microenvironment, cancer therapy, nanobiomaterials

## Abstract

The Hedgehog (Hh) signaling pathway is pervasively involved in human malignancies, making it an effective target for cancer treatment for decades. In addition to its direct role in regulating cancer cell attributes, recent work indicates that it has an immunoregulatory effect on tumor microenvironments. An integrated understanding of these actions of Hh signaling pathway in tumor cells and tumor microenvironments will pave the way for novel tumor treatments and further advances in anti-tumor immunotherapy. In this review, we discuss the most recent research about Hh signaling pathway transduction, with a particular emphasis on its role in modulating tumor immune/stroma cell phenotype and function, such as macrophage polarity, T cell response, and fibroblast activation, as well as their mutual interactions between tumor cells and nonneoplastic cells. We also summarize the recent advances in the development of Hh pathway inhibitors and nanoparticle formulation for Hh pathway modulation. We suggest that targeting Hh signaling effects on both tumor cells and tumor immune microenvironments could be more synergistic for cancer treatment.

## 1 Introduction

The Hedgehog (Hh), an evolutionarily conserved signaling pathway, regulates tissue homeostasis, regeneration, and tumorigenesis (1). During development, Hh signaling is significantly activated, and inadequate Hh signaling leads to a variety of developmental disorders, such as birth defects, cyclopia, and holoprosencephaly (2). In the adult mammals, Hh activity declines but becomes reactivated in tumor and tissue repairing contexts. Accumulating evidence indicates that abnormal activation of Hh signaling has been implicated in multiple aspects of tumorigenesis, including tumor initiation, progression, drug resistance, and metastasis (1, 3, 4). In recent years, emerging evidence has highlighted the immunoregulatory effects of the Hh signaling pathway in many malignant tumor microenvironments, which are established by tumors consisting of diverse nonneoplastic cells, including cancer-associated fibroblasts and immune cells such as macrophages and T cells (5). It has been shown that Hh signaling pathway modulates the activity and function of immune and stromal cells while increases programmed cell death ligand 1 (PD-L1) expression. Not surprisingly, the development of Hh inhibitors for targeted cancer therapy has attracted much attention. An improved understanding of the effects of Hh signaling on tumor cells, nonneoplastic cells and their complex crosstalk will pave the way for the development of novel tumor therapeutic strategies and further advances in antitumor immunotherapy. Here, we focus on the emerging roles of Hh signaling in tumor microenvironments, summarize the recent advances in Hh-targeting inhibitors and nanobiomaterial strategy for inhibiting Hh pathway as cancer therapy and deliberate the immunotherapeutic implications against Hh pathway.

## 2 Overview of Hedgehog signaling pathway

**Figure 1:**
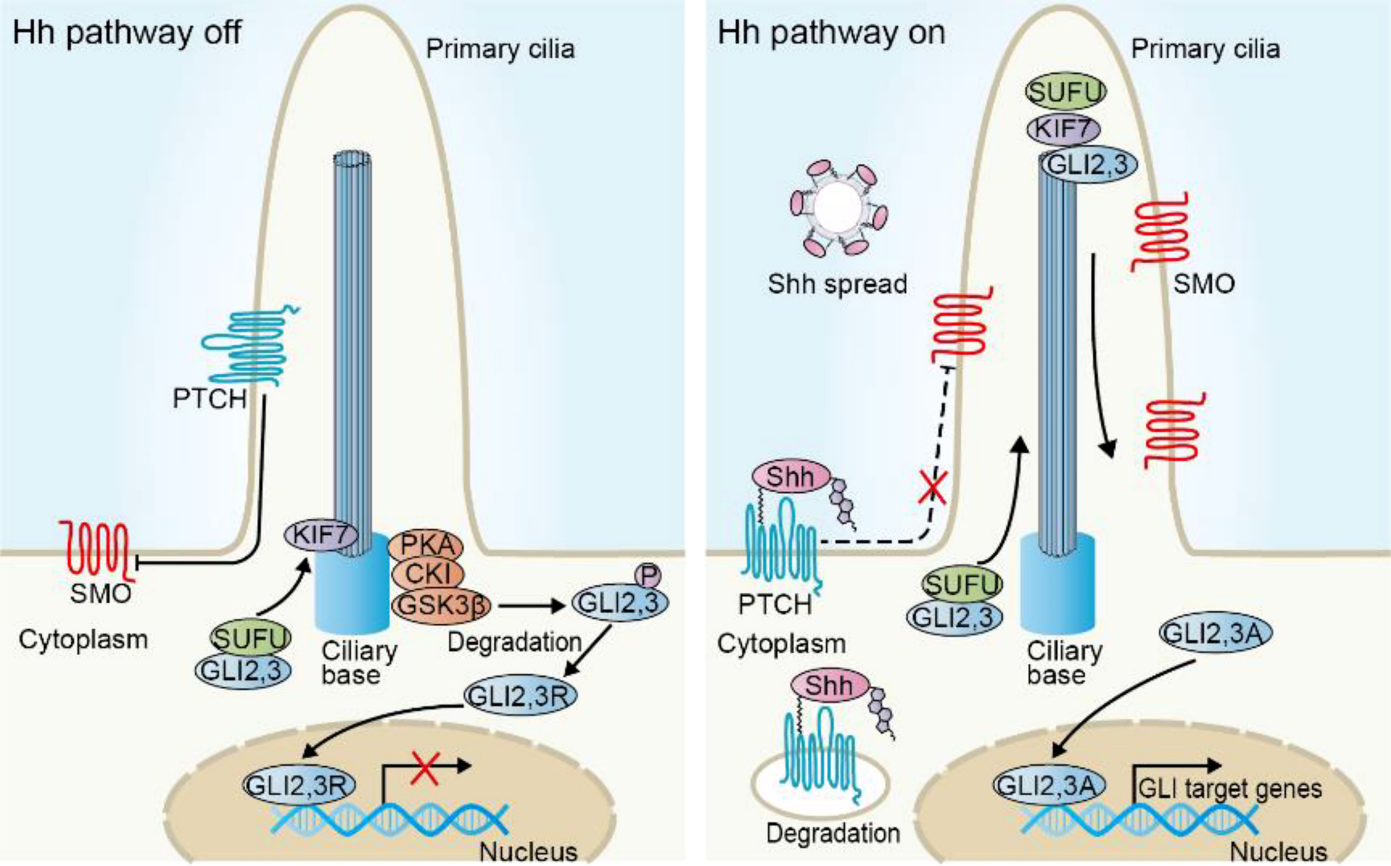
Overview of the Hh signaling pathway in mammalian cells. In the absence of Hh ligands, PTCH prevents SMO activity and cilia translocation. Transcription factors GLI2 and GLI3 are sequestered *via* binding with SUFU and phosphorylating by PKA, CKI and GSK3β to generate repressor forms (GLI2, 3R) that enter into the nucleus and inhibit the transcriptional program. Upon Hh ligands such as Shh, binding with PTCH, both of them are degraded in the cytoplasm. SMO accumulates in the cilia, where SUFU/GLI complex separates. The activator forms of GLI (GLI2, 3A) enter into nucleus and induce the transcription of target genes.

Hh signaling pathway was discovered 40 years ago in Drosophila melanogaster through massive gene screening (6). In mammals, the major components of Hh cascade contain secreted Hh ligands [Sonic Hedgehog (Shh), Desert Hedgehog (Dhh), and Indian Hedgehog (Ihh)], two transmembrane proteins [Patched (PTCH) and Smoothened (SMO)], and GLI transcription factors (GLI1, GLI2, GLI3) (7). Moreover, the primary cilia (PC), a tubulin-scaffold protrusion of the cell membrane, provides a specific interface for Hh signaling transduction by dynamically coordinating signaling proteins in this organelle (8). In this section, we will provide an overview and updates on the molecular mechanisms of Hh signaling transduction (**Figure 1**).

### 2.1 Hh ligands

The canonical Hh signaling is initiated by three homologous Hh ligands (Shh, Dhh, and Ihh), which have similar functions but are expressed in tissues with spatially and temporally varying patterns (9, 10). Nascent Hh proteins are palmitoylated on their amino-terminal domain (N-terminal) and cholesteroylated on the carboxyl-terminus (C-terminal), making them with high-affinity to cell membrane. With the help of transmembrane proteins including Dispatched (DISP) and vertebrate-specific SCUBE2, modified Hh proteins are released from the surface of secreting cell (11–13). Hh spreads with lipoprotein particles, extracellular vesicles or filopodial structure, which function as binding carriers and synergistically mediate Hh transportation from secreting cells to distant parts in the tissues, thus forming a functional morphogen gradient (14–18).

### 2.2 PTCH

PTCH, the primary receptor of Hh, is a pathway-suppressor protein. Structurally, PTCH is composed of two extracellular domains that bind Hh ligands, 12-transmembrane (TM) helices, and cytoplasmic carboxyl-terminal region (19, 20). Hh grasps and locks the extracellular domains of PTCH with its N-terminal palmitate and C-terminal cholesterol, thus blocking PTCH transforming into an inhibitory conformation (19, 21). Subsequently, the Hh/PTCH complex departs from the primary cilia and undergoes ensuing transportation into endosomes and degradation, thereby alleviating pathway inhibition (22). In the absence of Hh, PTCH is anchored on the cilia membrane and blocks SMO activity. One possible mechanism for this effect seems to be associated with PTCH-mediated blockade of contact between SMO and cholesterol, a putative endogenous activator (23).

In addition to PTCH, several other cell surface co-receptors have also been found to be involved in modulating Hh signal reception (24). For example, Interference Hedgehog (Ihog), CDON, BOC, and GAS1 synergistically bind Hh ligands and promote signaling activity, whereas vertebrate-specific Hh-interacting protein 1 (HHIP1) functions as a negative receptor.

### 2.3 SMO

SMO, a member of G protein-coupled receptor (GPCR) superfamily, consists of an extracellular cysteine-rich domain (CRD), a 7-transmembrane domain (7-TMD), and an intracellular C-terminal domain (25–27). In the context of insufficient Hh, PTCH antagonizes SMO activity (26). Given that PTCH does not physically interact with SMO, how PTCH regulates SMO activity remains enigmatic (28). Currently, the more accepted hypotheses hold potential second endogenous messengers involved in signaling communications between PTCH and SMO. Independent evidence puts forward cholesterol and endocannabinoids as possible candidates, which function as potent pathway activators or inhibitors, respectively (23, 29, 30). Cholesterol may be transported by a channel in PTCH from inner leaflet to outer leaflet membrane. Upon binding to PTCH, Shh occupies the channel and impedes this transportation, allowing sufficient inner leaflet cholesterol directly binding to the CRD of SMO, which induces SMO cilia accumulation and SMO-mediated signals (19, 31–33). This process is essential for full activation of Hh, since cholesterol deficiency, SMO CRD depletion or mutation in key residues that dampen cholesterol binding, impairs SMO activity (23, 34–36). Moreover, with biochemical fractionation and lipidomics, Eaton et al. identified lipoprotein-derived endocannabinoids as potential endogenous SMO inhibitors (29). Cannabis consumption during pregnancy induces holoprosencephaly and ventral neural tube patterning defects in *Cdon* mutation mice and links to human birth defects, which mimicked Hh inhibition phenotype *in vivo* (37; 38; 39). However, whether or not additional endogenous molecules coordinate PTCH/SMO transduction, how PTCH regulates membrane cholesterol and how cells sense these lipids are still in question.

Hh signaling promotes PTCH to exit PC, where cholesterol-bound SMO accumulates. This process is likely to be associated with the co-localization of downstream components at PC tip, which is essential for signal transduction.

### 2.4 SUFU

Suppressor of fused (SUFU) acts at the level between SMO and the final effector GLI. SMO accumulation in cilia induces an inactive SUFU-GLI complex to the tip of cilia, where this complex undergoes dissociation, allowing GLI activation (40, 41) (**Figure 1**). Recent studies revealed the complex modulation mechanism of SUFU for Hh activity. SUFU can directly interact with GLI proteins and keep them in the cytoplasmic localization, thereby inhibiting transcriptional activity (42, 43). GLI can also be bound by SUFU in the nucleus, which impedes the recruitment of transcriptional coactivator and promotes transcriptional repressor interaction, inhibiting Hh target gene expression (44, 45). Unexpectedly, the most recent study has revealed its positive effect on GLI activity in some cases (46). In a substantially higher level of GLI2 context, such as in *Smo* activation, SUFU inhibits SPOP-mediated GLI2 proteasomal degradation to stabilize GLI2, meanwhile, SUFU serves as a transcriptional target of GLI2, thereby developing a positive feedback loop and increasing Hh activity. In line with this, mice with conditional *Sufu* deletion in robust GLI2 expression background display reduced GLI activity and have significantly prolonged survival of medulloblastoma-prone mice, indicating the biphasic and contextual roles of SUFU with regard to tumor formation (46).

### 2.5 GLI

The transduction of Hh signaling needs to activate its downstream GLI transcription factors. In mammals, there are three homologous GLI proteins: GLI1, GLI2, and GLI3. Structurally, GLI2 and GLI3 contain C-terminal activator domain and N-terminal repressor domain, whereas GLI1 only has a C-terminal domain. In this regard, GLI1 functions as a transcription activating factor and itself serves as the target gene of Hh pathway, hence positively amplifying the activated responses, while GLI2 and GLI3 form full-length activator, or when cleaved off into truncation, suppressor form. Hh input signals change the ratio between the activator and repressor forms, influencing target genes (1). In the absence of upstream Hh signals, GLI2 and GLI3 are phosphorylated by protein kinase A (PKA), glycogen synthase kinase 3β (GSK3β) and casein kinase 1a (CK1a), resulting in ubiquitin-dependent proteasome degradation, in which C-terminal activator domain is removed and the remaining protein containing GLI repressor forms (GLI R) enters into the nucleus, ultimately repressing gene transcription (47). When activated, phosphorylation and ubiquitination of GLI are suppressed; full-length GLI is maintained in nucleus to evoke the transcription of target genes, including genes associated with proliferation (*Cyclin D1*, *MYC*), apoptosis (*BCL2*, *MCL1*), epithelial to mesenchymal transition (*SNAIL*), angiogenesis (*VEGF A* and *B*), Hh pathway feedback (*GLI1*, *PTCH1*, *HHIP*), and stemness (*NANOG*, *SOX2*). Given that GLI3, compared with GLI2, is more efficiently processed into repressor form (47), GLI2 seems to be the primary transcription factor in charge of activating Hh signaling, while GLI3 exerts the suppressive function.

### 2.6 Non-canonical activation of Hedgehog signaling pathway

Hh signaling transduction cascade described above refers to canonical Hh pathway in a ligand/receptor-dependent manner. However, there is also a growing body of evidence to support the non-canonical activation mechanisms in tumors. These include, but are not limited to, SMO- or Shh-independent GLI activation; GLI-independent activation yet SMO-dependent or PTCH-dependent mechanisms (48). For example, SMO has been demonstrated to couple with heterotrimeric G proteins regulating cytoskeleton, which is independent of GLI transcriptional activity. This cytoskeletal change is essential for Hh-mediated cell migration and tubulogenesis in endothelial cells (49, 50). Our group recently demonstrated that prostaglandin E2-induced activation of c-Jun N-terminal kinase (JNK) increased GLI2 expression in a SMO-independent mechanism through phosphorylating GLI2 at Thr1546, which is important for colorectal cancer cell proliferation (51). These reports could partially explain the resistance mechanism of SMO inhibitors in clinic. Therefore, it will be worth elucidating the contribution of non-canonical signaling to tumors and developing novel pharmacological targeting and combinatorial treatment strategies. In-depth discussions about the role of non-canonical Hh signaling in tumor development have been reviewed elsewhere (48, 52–54).

## 3 Hedgehog signaling in cancer

**Figure 2:**
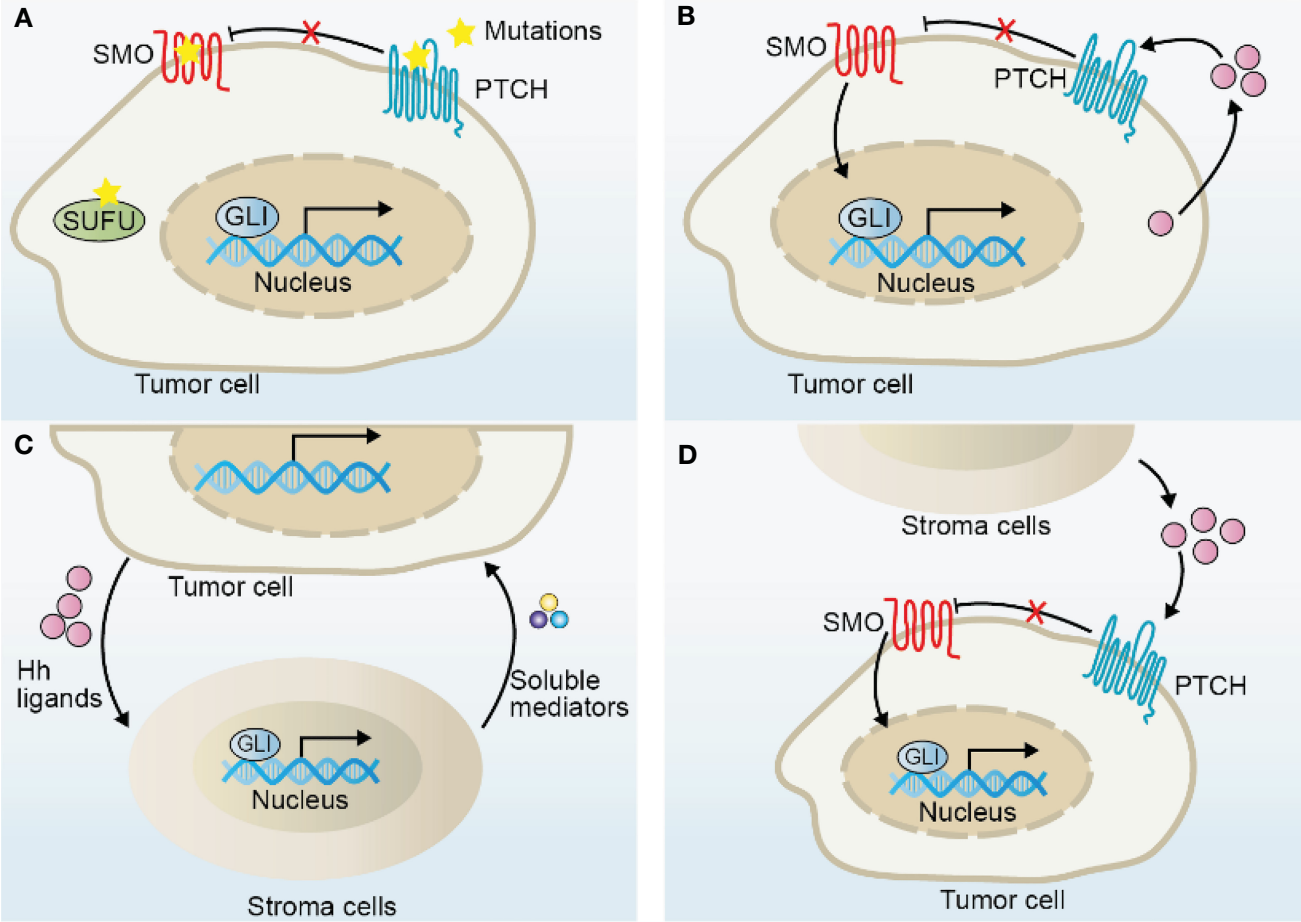
Models of Hh signaling pathway activation in cancer. **(A)** Hh ligand-independent activation due to the mutations of Hh components, including mutations in PTCH, SMO, SUFU, and GLI amplifications. **(B)** Hh ligand-dependent autocrine activation due to excessive Hh ligands secreted by tumor cells to induce Hh signaling in tumor cells themselves. **(C)** Hh ligand-dependent paracrine activation due to excessive Hh ligands secreted by tumor cells to induce Hh signaling in nonneoplastic cells, such as fibroblasts and macrophages, which in turn secrete soluble mediators, such as IL-6, TNF-α, and VEGF to promote tumor growth. **(D)** Hh ligand-dependent reverse paracrine activation due to Hh ligands produced by stroma cells to induce Hh signaling in tumor cells.

Many cancers are associated with uncontrolled Hh signaling. In general, four modulatory models of Hh pathway in tumors have been described (**Figure 2**).

Hh ligand-independent tumor was first identified in patients, who harbor germline loss-of-function mutation in *PTCH1* (55), with nevoid basal cell carcinoma syndrome (also known as Gorlin syndrome), tending to develop tumors, especially basal cell carcinoma (BCC), medulloblastoma (MB) and rhabdomyosarcoma (56). Oncogenesis in this type is caused by mutations in Hh pathway components, which confer unscheduled Hh activation and cell-intrinsic growth characteristics (**Figure 2A**). Almost all sporadic BCC and more than 30% cases of MB have been implicated in Hh mutational activation. The most common mutations in these tumors include loss-of-function mutations in *PTCH* and *SUFU*, gain-of-function mutation in *SMO*, or gene amplifications in *GLI1* and *GLI2*, as shown in mouse or human cancers, resulting in uncontrolled Hh activity and promoting tumorigenesis (57, 58). Consistently, mice with *Ptch1* mutation are susceptible to develop MB, especially in those with additional depletion in *p53* allele or in homozygous *ND2:SmoA1* (*Smo* activation mutation) transgenic mice, and develop UV-induced BCC (59–62), suggesting that Hh signaling pathway is a major driver in the development of these tumors. Mice with the epidermis-transgenic expression of *Gli1* or mice with *Sufu+/-* heterozygote develop a skin tumor phenotype resembling Gorlin syndrome (63, 64). Moreover, increased levels of GLI protein expressions are frequently associated with poor prognosis and therapeutic resistance in MB or BCC (65, 66). Remarkably, SMO inhibitors have been successful in treating patients with BCC in clinic. To be mentioned, mutations in Hh components, such as SUFU, have also been identified in other cancer types, such as prostate cancer (67), albeit less frequent, but potential mechanistic relevance in tumorigenesis is unclear.

Hh ligand-dependent mechanisms have also been reported in a variety of tumors, where Hh ligands produced by tumor cells actively promote Hh signaling activity in the tumor cells themselves (autocrine) or tumor microenvironment cells (paracrine) (**Figures 2B, C**). The key hints for functional relevance of Hh ligand-dependent mechanisms in tumorigenesis result from several independent studies. In contrast with ligand-independent tumors, although Hh activity is abnormally activated, these tumors do not harbor Hh component mutations and their pathway activity largely depends on Hh ligands (68). Besides, many tumors express abundant levels of Hh ligands, especially Shh and Ihh, such as pancreatic (69), colon (70), breast (71), bladder (72), gastric (73) and small cell lung (74) cancers. Early assumptions for the ligand-dependent autocrine models came from observations that Hh ligands drove the proliferation of tumors cells and Hh-neutralizing antibody dramatically blocked its pro-tumor effect *in vitro* (68), however the potential mechanism is elusive since these tumor cells did not display a corresponding decrease in Hh target gene expressions, demonstrating the existence of alternative assumptions. Indeed, a recent study demonstrated that Shh induced proliferation at least in part due to the inhibition of PTCH-mediated apoptotic activity, leading to compromised apoptosis of tumor cells (75).

Hh-dependent paracrine mechanism may also be involved in tumorigenesis. In multiple Hh ligands-expressing human tumor xenograft models, Hh target gene expressions specifically increased in tumor-infiltrating nonneoplastic cells rather than tumor cells, indicating the activated Hh signals in tumor microenvironment cells, far from tumor cells (76).In line with this, recent single-cell RNA-sequencing data from pancreatic ductal adenocarcinoma (PDAC) patient-derived tissues and organoids revealed that Hh ligands are confined to tumor epithelial cells, whereas Hh target gene expressions were largely restricted to stromal cells (77–80), furtherly supporting the notion of ligand-dependent paracrine mechanisms. In this model, tumor microenvironment cells in turn secrete soluble mediators, such as IL-6, TNF-α and VEGF to promote tumor growth (81). Notably, updated research has demonstrated the elusive role of Hh in paracrine-dependent tumors. Blocking Hh activity may not always restrain tumor growth, but may accelerate tumor progression even in some circumstances. For instance, in xenograft PDAC tumor models, pharmacological inhibition of Hh potentially reduced tumor growth along with a reduction of Hh activity in the mouse stroma cells (76, 82). Surprisingly, more recent studies also observed that *Shh* deletion in the pancreatic epithelium, *Smo* deletion from stromal fibroblasts or chronic Hh inhibition using a SMO inhibitor accelerated PDAC progression and shortened survival, suggesting its tumor suppressor roles (78, 79, 83). The tumor-restraining functions of paracrine Hh (mainly refers to Shh/Ihh) have also been demonstrated in other tumors, such as colon cancer (84), bladder cancer (85), prostate cancer (86) and lung adenocarcinoma (87). These seemingly paradoxical results appear to be associated with the heterogeneity of fibroblasts, which we will discuss in the next section. To summarize, the two Hh-ligand dependent hypotheses are not mutually exclusive and tumors may take advantage of both signal transduction models to optimize growth.

Interestingly, Hh ligand-dependent reverse paracrine signaling has also been observed in tumors (**Figure 2D**). In this case, Hh ligands derived from microenvironment cells provoke Hh signaling activity in tumor cells (88, 89). For example, malignant lymphoma and plasmacytoma cells receive Hh signaling from bone-marrow, nodal and splenic stromal cells, thereby enhancing malignant process (88).

## 4 Hedgehog signaling pathway modulates tumor immune microenvironment

**Figure 3:**
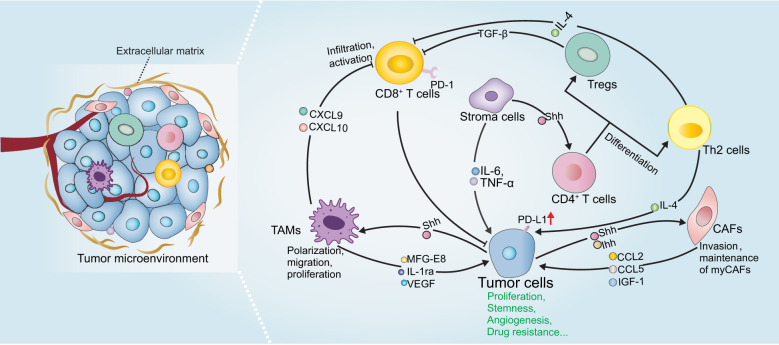
The role of Hh signaling in tumor immunity. Shh derived from tumor cells or stroma cells activates Hh signaling pathway in TME cells and regulate the phenotype and function of TAMs, CAFs and T cells, which in turn secrete various immunomodulatory factors and many other soluble mediators to promote tumor malignant progression. Please refer to the main text for details.

The tumor microenvironment (TME) serves as a crucial contributor to tumorigenesis, malignant development, metastasis, and drug resistance (90, 91). The TME is composed of heterogeneous cancer cells, immune cells, cancer-associated fibroblasts (CAFs), and cancer stem cells along with extracellular components, such as cytokines, chemokines, and extracellular matrix (ECM). All of these contents interact with each other to develop an immune escape milieu. Especially, the component cells in the TME exert distinct functions to modulate tumor immunity. Over the past few years, immunotherapy has become a hot pot in basic, translational, and clinical research. Despite of its great power in cancer treatment, the emerging resistance to immunotherapy cast a shadow over this field. Thus, it is important to comprehensively understand immunosuppressive mechanisms within TME to develop rational combination therapy aiming to improve or overcome the resistance to immunotherapy. Moreover, ongoing studies provide novel insights into the potential of Hh pathway in regulating immune responses in several malignant neoplasms. As the immunomodulatory roles of Hh pathway are scarcely discussed, in this part, we mainly summarize the latest findings about Hh pathway in the regulation of the tumor-associated macrophages, T cells, CAFs, and immune checkpoint PD-L1 (**Figure 3** and **Table 1**).

**Table 1:** The functions of Hh signaling in regulating TME and the potential mechanisms.

Effector TME cells/checkpoint	Biological functions	Mechanisms	Cancer/Disorder models	References
Tumor cells	Increased proliferation, migration, drug resistance and stemness	Activated Hh signaling pathway	Various tumors	(92)
Macrophages	Induced M2 polarization and reduced inflammatory responses	Regulated the transcription of KLF4, IL-4 and promoted metabolic reprogramming	Hepatocellular carcinoma, lung carcinoma, mammary tumor, multiple myeloma	(93–97)
	Increased proliferation	Enhanced BMI1 transcription	Multiple myeloma	(97)
	Enhanced migration	Upregulated chemokine receptor CCR2	Gastric epithelial regeneration	(98)
T cells	Promoted Th2 transcriptional programs and differentiation	Positively regulated the transcription of IL-4	Allergic disease	(99)
	Promoted Treg differentiation	Upregulated the expressions of FOXP3 and TGF-β	Atopic dermatitis	(100)
PD-L1	Induced PD-L1 expression in tumor cells on hypoxia context	Not determined	Multiple cancer cells *in vitro*	(101)
	Induced the transcription of PD-L1 in tumor cells	GLI1 and GLI2 directly bound to the PD-L1 promoter	Human gastric cancer organoid	(102, 103)
	Induced the transcription of PD-L1 in TAMs	Participated in STAT3-mediated PD-L1 induction	Hepatocellular carcinoma, lung carcinoma	(96)
	Induced PD-L1 expression in dendritic cells	Induced PD-L1 expression at post-transcriptional level by blocking miR-324-5p and miR-338-5	Mycobacterial infection	(104)
Fibroblasts	Promoted myCAF invasion	Not determined	Pancreatic cancer	(82)
	Maintained myCAF subtype	Hh inhibition reduced myCAF numbers and increased iCAF numbers, which promoted to develop immunosuppressive microenvironment; Ihh inhibited the secretion of CXCL12 and thus attenuated immune cell infiltration	Pancreatic cancer; colon	(80, 105)
	Fueled malignant phenotype of tumors	Induced the secretion of soluble mediators, such as IGF-1, Activin, lactate to enhance Hh signaling in tumor cells	Multiple tumor types	(106–108)

### 4.1 Hedgehog signaling pathway drives the polarization and proliferation of tumor-associated macrophages

Tumor-associated macrophages (TAMs) are a population of heterogeneous myeloid-derived immune cells in the TME, which display pro- or anti-tumor functions that influence tumorigenesis (109). TAMs are generally polarized into two subtypes, including type I macrophages (M1), which are proinflammatory and contribute to inhibiting tumor development, and type II macrophages (M2), which are immunosuppressive contributing to protecting tumors from immune surveillance and pro-angiogenesis. The signaling cues from TME are able to regulate macrophage polarization and function. In most cases, TAMs tend to assume M2 phenotype. Increased number of infiltrating M2-like TAMs is often associated with resistance to immune checkpoint therapies and shorter survival among patients with various cancers (110–115). Thus, drugs that can inhibit M2-like TAM phenotype and induce M1-like phenotype may be beneficial for tumor therapy.

Recent studies have highlighted the importance of Hh in regulating the phenotype and function of TAMs, both of which are critical for tumor immune responses. Tumor-derived Shh has been shown to facilitating M2 polarization of TAMs in hepatocellular carcinoma, lung carcinoma, mammary tumors and multiple myeloma *in vivo* and *in vitro* (93–97). Accordingly, pharmacological or genetic inhibition of *Smo* in myeloid cells (including macrophages, monocytes, and granulocytes) or Shh-deletion in tumor cells dramatically decreased M2 polarization and tumor growth. Importantly, loss of Hh activity in TAMs was associated with enhanced CD8^+^ T cell infiltration in the TME by the suppression of chemokines CXCL9 and CXCL10, and CD8^+^ T cell effector functions by promoting PD-L1 expression *via* STAT3, thereby influencing the survival and tumor development in hepatoma, indicating that Hh modulates T cell inhibition at least in part by TAMs in Hh-ligand dependent paracrine tumors. Distinct mechanisms of Hh-induced TAM polarization have been proposed, including GLI-triggered transcriptional regulation of Krüppel-like factor 4 (KLF4) and IL-4 (93, 94), as well as metabolic reprogramming, such as O-GlcNAcylation biosynthesis, lipid metabolism and mitochondrial adaptations (95). Notably, combining a Hh inhibitor with macrophage depletion may potentially improve the therapeutic benefits, providing a probable combination strategy in clinical trials for patients with Shh-overexpression cancers. Since Shh is also highly expressed in other cancer types, such as colorectal cancer, pancreatic adenocarcinoma and gastric cancer (116), it deserves exploring its novel immunotherapeutic potential in these tumors in the future. In addition to the effect on differentiation status of TAMs, a more recent study demonstrated that Hh activity also influenced the proliferation of TAMs in multiple myeloma (97). Mechanistically, Hh signaling positively regulates *BMI1* transcription, leading to TAMs proliferation, which drives angiogenesis by upregulating VEGF, NO expression and thus contributing to their pro-myeloma functions (97). Moreover, Jayati and colleagues identified Shh as a chemoattractant to induce macrophage migration by upregulating the expression of CCR2, a crucial receptor expressed in macrophages for chemotaxis, *via* SMO-dependent mechanism during gastric epithelial regeneration (98).

On the other hand, macrophages may positively regulate Hh activity during tumor initiation and progression by producing soluble mediators. Jinushi et al. clarified that TAMs-derived milk-fat globule-epidermal growth factor-VIII (MFG-E8) activated Hh signaling pathways in cancer stem cells, and induced tumorigenicity and anticancer drug resistance (117). Besides, Wang and colleagues also showed that IL-1ra production by macrophages enhanced tumor cell stemness and metastasis *via* activating Hh pathway (118). Moreover, LPS-activated M1 macrophages were able to produce Shh, which furtherly promotes the stem cell phenotype, consequently mediating tumor growth and chemoresistance (89).

### 4.2 Hedgehog signaling pathway controls T cell activation and differentiation

Mature T lymphocytes are primarily divided into CD8^+^ or CD4^+^ T cells according to their molecular characteristics (119, 120). CD8^+^ T cells are the main effector cells that eliminate tumors and protect tumor cells from immune escape *via* secreting cell cytolytic molecules and pro-inflammatory cytokines. CD4^+^ T cells mainly regulate immune effects by secreting cytokines to enhance or dampen immune responses.

Growing evidence demonstrated the essential role of Hh signaling on T-cell fate by regulating tumor-antigen specific T cell receptors (TCRs) that recognize antigens and associate with the activation and resolution of T cells, in physiological conditions (121–123). Increased Hh activity diminished TCR signal strength, thus impairing the ability of TCR to transduce signals to T cells for activation and proliferation. Mechanistically, Hh activation compromised TCR-induced calcium influx and major components of the TCR signaling pathway, including AP-1, NF-κB and phosphorylated ERK (124, 125). The role of Hh signaling in T cells under pathological conditions has also been identified. T cell differentiation is affected by Hh signaling. CD4^+^ T cells with GLI2 constitutively activated tend to adapt Th-2 like profiles, which is characterized by secretion of IL-4 and IL-13, and this seems to be associated with GLI-mediated transcriptional upregulation of Th-2 cell master regulator IL-4, consequently enhancing allergic inflammation and inhibiting cytolytic CD8^+^ T cells in the context of tumors (99, 126, 127). Conversely, Hh-deficient CD4^+^ T-cells adopt Th-1 phenotype, which produces interferon-gamma (IFN-γ) and evokes CD8^+^ T cell activation and tumor suppression (99). Regulatory T cell (Treg) plays an immunosuppressive role by dampening the effector function of T cells. Interestingly, in mice with induced atopic dermatitis (AD), Hh signals to CD4^+^ T cells to trigger the upregulated expression of FOXP3 and TGF-β, which is crucial to Treg terminal differentiation and consequently reduce T-cell function, suggesting the anti-inflammatory action of Hh in AD (100). Since many cancers express excessive Hh ligands, we therefore, hypothesize that Hh might also signal to tumor local T-cells to skew immune response. Indeed, potential relevance between Hh and T cell activity has also been reported in tumor microenvironment. Tumors with high Hh levels tend to display lower fractions of CD8^+^ T cells and significantly enrich more Tregs in pan-cancer analyses (128, 129). Similarly, *Ptch*-mutation and *SmoM2* triggered BCC mouse models exhibited strong infiltration with Tregs and secretion of immunosuppressive signals, such as IL-10 and TGF-β in the tumor lesions (130, 131). In line with this, Hh pathway inhibitor induced tumor regression and accompanied by recruitment of CD4^+^ and cytotoxic CD8^+^ T cells and an upregulation of MHC class I according to IHC analysis of biopsies from patients with BCC, indicating the activation of immune microenvironment (132). Activated T cell after Hh inhibition was also observed in breast cancer, hepatoma, and Lewis lung carcinoma (93, 94, 96). However, whether of the intrinsic role of Hh in T cells also plays an immunosuppressive role in the tumor microenvironment is largely unknown and remains to be investigated in the future.

### 4.3 Hedgehog signal induces PD-L1 expression

The immune checkpoint receptor PD-1 (also known as CD279) and its ligand PD-L1 (also known as CD274) represent the dominant immune checkpoint pathway. In the TME, PD-1 is mainly expressed by lymphocytes, whereas PD-L1 has been detected on several cell types, including tumor cells, fibroblasts, TAMs and lymphocytes. Engagement of PD-1 with PD-L1 prevents T cell proliferation, activation and cytokine production, thus blockade of PD-1/PD-L1 interactions serves as a promising strategy for cancer therapy (133).

Several studies report on the associations of Hh signaling for modulating PD-L1. A recent pan-cancer analysis of gene signature demonstrated that tumors with comparatively higher Hh activity, especially in uterine corpus endometrial carcinoma, skin cutaneous melanoma and ovarian serous cystadenocarcinoma, display upregulated PD-L1 and relatively fewer immune effectors (129), indicating possible links between Hh signaling and PD-L1-mediated tumor immune responses. Further supporting this notion was that the expression of GLI2 has a positive correlation with PD-L1 and negatively correlated with infiltrating CD8^+^ T cell in gallbladder cancer and gastric cancer tissues (103, 134). *In vitro*, hypoxia-induced PD-L1 expression was shown to be at least partially dependent on Hh signaling in various cancer cells, subsequently skewing the lymphocyte activation (101). Also, in human gastric cancer organoid models, Hh signaling induced PD-L1 expressions, and GANT-61, a Hh inhibitor targeting GLI, significantly downregulated PD-L1 and evokes anti-tumor immune (102, 103). At the molecular level, GLI1 and GLI2 can directly bind to the PD-L1 promoter to promote its transcription, which is independent of SMO (103), suggesting that targeting GLI may gain more favorable outcomes in this case.

In addition to regulating PD-L1 expression in cancer cells, Hh signaling also has a role in modulating PD-L1 in immune cells. STAT3 represents one of the major transcription factors that induce PD-L1 expression (135). In TAMs, GLI1 directly binds to the promoter of STAT3 and induces its expression, facilitating PD-L1 transcription and thereby leading to suppression of CD8^+^ T cell effector functions. In line with this effect, deletion of *Smo* in myeloid cells significantly blocked PD-L1 expression and increased effector functions of CD8^+^ T cell as measured by IFN-γ and granzyme B secretion and reduced tumor growth (96). Furthermore, GLI1 also facilitated PD-L1 expression by suppressing its negative regulators miR-324-5p and miR-338-5 in human DCs, leading to the expansion of Tregs (104). Together, Hh signaling serves as a stimulator of PD-L1 expression in multiple cell types, raising the possibility that Hh activity seems to be a predictive biomarker for anti-PD-1 therapy and a rational combination of checkpoint inhibitors with Hh inhibitors would gain more therapeutic advantages than monotherapy.

### 4.4 Hedgehog signaling pathway regulates interactions between cancer cells and cancer-associated fibroblasts

CAFs are one of the most abundant cell populations in TME, which is tightly involved in tumor immune evasion, cancer metastasis and angiogenesis by synthesizing ECM and secreting various immunomodulatory effectors and growth factors (136, 137). Recent reports revealed their phenotypic and functional heterogeneity across/within tumors. For example, several CAF subsets have been identified in PDAC *via* single-cell RNA sequencing, such as myofibroblastic CAFs (myCAFs) and inflammatory CAFs (iCAFs) in TME, wherein myCAFs characterized by high α-SMA expression, contribute to secreting ECM proteins, tumor migration and resistance, whereas iCAFs secrete inflammatory factors, such as IL-6, IL-11 (138). The existence of CAF subpopulations with immunomodulatory functions has also been identified in breast cancer and melanoma (CAF S1)(139, 140). Functionally, most of CAFs in TME promote tumor development, while tumor-suppressing CAFs have also been identified, indicating the biphasic roles in TME. Changes in the compositions of CAFs may cause distinct outcomes (137, 138, 141).

Knowledge in the correlation between Hh and CAFs is currently emerging. CAF infiltration and fibrous tissue deposition are positively correlated with Hh activity, indicative of biologically relevant functions (142). Intriguingly, the functional role of Hh signaling in CAF-mediated tumor development seems pleiotropic, and modulation of Hh signaling in CAFs may lead to contradictory results. In PDAC tumor xenograft models, acute inhibition of Hh reduced tumor-associated ECM and improved sensitivity to chemotherapy, thereby providing a survival advantage *in vivo* (143, 144). However, recent studies have challenged this dogma. In Kras-mutated (G12D) mouse models, CAFs with *SMO* depletion resulted in pancreatic acinar-ductal metaplasia and subsequently promoted the initiation of PDAC (83). Hh inhibition with genetic deletion of Shh or chronic SMO inhibitors reduced myCAFs and accelerated PDAC progression (79, 85). A disappointing response of patients with PDAC to Hh inhibitors has also been observed in clinic (79, 145–147). These results potentially indicate that Hh signaling may regulate different CAF subsets to execute tumor-restricting or tumor-promoting functions. Indeed, single-cell RNA sequencing clarified the distinct activation state of Hh signal in CAF subsets, of which myCAFs displayed higher Hh pathway activity and are preferentially activated compared with iCAFs in mouse and human PDAC. Long-term Hh pathway inhibition resulted in myCAF depletion and iCAF enrichment, which contributed to a more immunosuppressive microenvironment and disease progression (80), indicating the importance of selectively targeting CAF subpopulations, but not pan-CAF ablation as previous description. Although, the tumor-restricting role of Hh-mediated CAF has also been reported in other tumors, such as colon cancer, bladder cancer (84, 85), the specific functions of Hh pathway in different CAF subpopulations are not clear and remain to be identified. Furthermore, paracrine Hh ligands also promotes CAFs to produce factors, such as IGF-1, activin, lactate, which in turn fuel malignant phenotype of tumor cells (106–108).

Reciprocally, CAFs also activate Hh signaling in cancer cells by producing soluble mediators, such as CCL2, CCL5 and IGF-1. All of them can promote tumor progression in many aspects, including enhanced proliferation, migration and stem cell phenotype (148, 149). In patient-derived xenografts colorectal cancer models, CAFs-secreted growth factor TGF-β2 directly induced transcription of GLI2, thereby contributing to increasing stemness and intrinsic resistance to chemotherapy (150). Pharmacological inhibition of Hh and TGF-β2 synergistically enhanced the effect of chemotherapies. Furthermore, CAFs-derived exosomes cargo Shh promoted proliferation and malignant progression of esophageal squamous cell carcinoma and these effects can be partially mitigated by Hh inhibitor (151). Thus, Hh signaling has non-negligible effects on the crosstalk between CAFs and tumor cells, which is crucial for cancer therapy.

In summary, Hh signaling pathway not only directly promotes the proliferation and survival of tumor cells, but also induces the secretion of various immune suppressive cytokines, upregulates PD-L1 expression to develop an immunosuppressive microenvironment, and mediates the tumor-restricting or tumor-promoting functions of CAFs (**Table 1**). Thus, proper modulation of Hh pathway may represent reciprocal benefits for combating tumor growth and reverting pro-tumor TME.

## 5 Targeting Hedgehog signaling for antitumor therapy

Hh signaling has emerged as an attractive target in anticancer drug development. In this part, we will review the latest advances of Hh pathway inhibitors, with a focus on tumor immune therapy.

### 5.1 Small molecule inhibitors targeting at Hedgehog pathway

In the last few decades, great efforts have been devoted by pharmaceutical companies and academia to design new Hh inhibitors and several central Hh pathway components have been proved to be potential drug targets, mainly including Hh ligands, SMO and GLI proteins (**Table 2**).

**Table 2:** Compounds discussed in this review.

Compounds	Targets	Models of action	References
5E1	Shh	Shh monoclonal antibody	(152, 153)
MEDI-5304	Shh, Ihh	Shh and Ihh monoclonal antibody	(154)
robotnikinin	Shh	Binds to Shh	(155)
HL2-m5	Shh, Ihh, Dhh	Binds to Shh, Ihh and Dhh with similar affinity	(156)
berberine	Shh, SMO	Promotes Shh mRNA degradation and targets SMO with unclear mechanisms	(157, 158)
RU-SKI	Shh	Inhibits Shh palmitoylation	(159)
cyclopamine	SMO	Binds to the 7-TMD and CRD of SMO and induces its cilia localization	(160)
IPI-926 (saridegib)	SMO	Cyclopamine analogue	(161)
GDC-0449 (vismodegib)	SMO	Binds to SMO 7-TMD	(162–164)
LDE-225 (sonidegib)	SMO	Binds to SMO 7-TMD	(165, 166)
PF-04449913 (glasdegib)	SMO	Binds to SMO 7-TMD	(167, 168)
ABT-199	SMO/SUFU	Targeting SMO CRD/Disturbing SUFU-GLI interactions	(169, 170)
MIMX, ABT-263	SUFU	Disturbing SUFU-GLI interactions	(169)
SIAIS361034	SUFU	Disturbing SUFU-GLI interactions	(171)
GANT-61/GANT-58	GLI	Binds to GLI1 and GLI2 to prevent their binding with DNA	(172, 173)
GlaB	GLI	Binds to GLI1 to prevent its binding with DNA	(174)
arsenic trioxide	GLI	Binds to GLI1 and interferes with the stability of GLI2	(61, 175)
prostaglandin E1	GLI	Promotes the ubiquitin-mediated degradation of GLI2 *via* cAMP-PKA axis	(176)
TSA	GLI	Modulate GLI deacetylation by targeting HDAC	(177)
JQ1	GLI	Inhibits GLI1 and GLI2 transcription by targeting BRD4	(178)
Compound 25	GLI	Inhibits GLI1 and GLI2 transcription by targeting BRD4	(179)

Owing to increased production of Hh ligands by many cancers, disrupting the engagement between Hh and PTCH provides an available way to inhibit Hh signaling and curtail tumor growth. Hh neutralizing antibodies, including 5E1 (152, 153), a Shh monoclonal antibody, and MEDI-5304 (154), a Shh and Ihh monoclonal antibody, and small molecule compounds, including Robotnikinin and HL2-m5 (155, 156), have been reported to disrupt Hh/PTCH interactions, thereby suppressing Hh pathway and exhibiting anti-tumor activity. Shh transcription is also a potential targeting approach. Our previous research identified that natural product berberine significantly inhibited Shh expression at transcription level and delayed the growth of Shh-paracrine colon cancer *in vivo*, but the potential mechanism remains to be determined (158). Besides, interfering Hh ligand stability is another promising strategy. For example, RU-SKI has been reported to interfere Hh acyl-transferase-regulated Hh palmitoylation, which is an essential process for Hh protein stability and activity (159). However, one should notice that this type of Hh inhibitors might only display therapeutic effects in ligand-dependent tumors.

SMO inhibitors represent the predominant antagonists of Hh signaling pathway. Cyclopamine, a steroidal alkaloid derived from *corn lilies*, was the first identified Hh antagonist by directly binding to SMO (23, 160, 180, 181). Owing to its poor solubility and moderate potency, it was just used as a tool compound, not for clinical development. Some derivatives of cyclopamine were developed with more potent pharmacologic properties, such as saridegib (aka IPI-926) by Infinity (161). Of all the specific SMO inhibitors, there are only three on the market. The “first-in-class” Hh inhibitor is vismodegib (also known as GDC-0449) from Genentech, and approved by FDA in 2012 for the treatment of metastatic, recurrent and locally advanced BCC (163, 164), followed with sonidegib (aka LDE-225) from Novartis for locally recurrent and advanced BCC in 2015 (165, 166). In general, these two drugs displayed comparable response rates in patients with locally advanced basal cell carcinoma (182). Currently, they are actively undergoing clinical trials for other solid cancers. In 2018, a great advance came with the FDA approval of another SMO inhibitor glasdegib (aka PF-04449913) for treatment of patients with acute myeloid leukemia (AML) (168). This orally administered drug combination with low-dose cytarabine (LDC) improved the overall survival duration to 8.8 months from 4.9 months compared with LDC treatment alone in AML (167). Those inhibitors against SMO discussed above almost all combine with the 7-TMD of SMO (**Table 2**). Recently, SMO CRD has also been identified as another binding domain(183–186). Since GDC-0449-resistance mutations predominantly lie within 7-TMD domain, compounds targeting at SMO CRD seems to have the potential, at least in part, to overcome the resistance of FDA-approved SMO inhibitors, thus providing an alternative strategy for inhibition of resurgent Hh activity. Our group has demonstrated that ABT-199 appears to target at SMO CRD and can inhibit Hh activity provoked by SMO mutations that do not respond to GDC-0449, *in vivo* and *in vitro* (170) (**Table 2**).

Loss of *SUFU*, GLI amplifications and alterations in alternative pathway, though less frequent than SMO mutations, have also been observed in bench and bedside trials (187). Hence, development of drugs targeting downstream of SMO aroused significant interest in this field (188). Previous study found that BCL2 families (BCL2, BCL-XL, MCL1) interact with SUFU, thus disturbing SUFU-GLI interactions and inducing GLI targeting genes (169). Pharmacologic BCL2 inhibitors suppressed GLI-associated tumor growth. Intriguingly, we recently reported a selective BCL-XL PROTAC degrader disabling BCL-XL/SUFU interaction and countering drug resistance, especially, without effect on the bone growth of young mice (171). Compared with SUFU inhibitors, more GLI antagonists have been identified. Cellular screening identified the first such inhibitors, GANT-61/GANT-58 which block GLI transcription by interfering GLI1- DNA binding and effectively suppress the growth of tumors with elevated GLI1 expression, such as Ewing’s sarcoma (172, 173). Due to the poor pharmacodynamics and pharmacokinetics, they are just widely used as a tool. Similar to GANT-61, with virtual screening and structure docking leads to the discovery of GlaB, which directly engages GLI1 and thus blocks GLI1-DNA binding (174). Remarkably, arsenic trioxide, an FDA-approved drug for acute promyelocytic leukemia, has also been reported to act at the level of GLI by not only disturbing the stability and ciliary translocation of GLI2 but also directly binding to GLI1 (61, 175). Arsenic trioxide effectively suppressed tumors with abnormal Hh activation, such as primary medulloblastoma, vismodegib-resistant medulloblastoma and Ewing sarcoma *in vivo* when used alone or in combination with SMO inhibitor (61, 175, 189). It has been used in clinic for many years with clear safety-profiles, thereby providing a strong opportunity for clinical trials. On the other hand, in addition to direct GLI inhibitors discussed above, developing indirect GLI antagonists is well appreciated, since the transcriptional effector GLI has a shallow structure and lacks drug binding pocket. For example, Prostaglandin E1, an FDA-approved drug, was reported to be a potential indirect GLI inhibitor by promoting phosphorylation and degradation of GLI2 *via* activating ubiquitin-proteasome pathway (176). Besides, epigenetically or transcriptionally targeting GLI is another strategy to inhibit GLI by affecting gene transcription. Histone deacetylases (HDACs) regulate transcription by removing the acetylation status of histones or transcription factors (190). HDAC1 and HDAC2 were identified as transcriptional enhancers of GLI activity by deacetylation. Pharmacological inhibition of HDAC using TSA inhibited Hh activity and the growth of Hh-dependent cerebellar granule cell progenitors *in vivo* (177). Furthermore, BRD4 has been identified to occupy *GLI1* and *GLI2* promoters to enhance GLI transcription (178). Hence, BRD4 inhibitors, such as JQ1 and compound 25 synthesized by our group, are able to essentially block GLI-mediated genes and Hh triggered tumors *in vitro* and *in vivo* (178, 179). Details of other compounds by antagonizing GLI can be found elsewhere (188, 191, 192). Of all GLI inhibitor, only arsenic trioxide is currently in clinical studies and has completed the phase II (NCT01791894) clinical trial. However, poor effect and adverse side effects have been reported (193).

### 5.2 Nanoparticle formulation for Hedgehog pathway modulation

In recent years, nanobiomaterial is emerging as a promising therapeutic strategy against multiple tumors. Nanoparticles (NPs) are capable of loading therapeutic drugs, small molecules and therapeutic peptides at intended target sites, such as tumor, and thus enhancing antitumor capabilities. Intriguingly, several studies have reported the beneficial of engineered NPs for modulating tumor microenvironment by encapsulating Hh inhibitors alone or in combination with cytotoxic agents.

Jun’s group designed a polymeric micelle nanoformulation (M-CPA/PTX) to co-deliver cyclopamine (CPA), which inhibited stroma-producing CAFs, and cytotoxic chemotherapy drug paclitaxel (PTX) to kill tumor cells (194). M-CPA/PTX significantly suppressed tumor growth and extended survival time, importantly, without influencing on collagenous matrix, which is key to restrain tumor growth and metastasis (79). This could indicate that reprogramming NPs would be a potential approach for targeting both TME and tumors with less adverse effects. Similarly, erythrocyte membrane-camouflaged PLGA nanoparticles (MNP)-encapsulated cyclopamine (CMNP) in combination with PTX-loaded MNP displayed more obvious tumor growth inhibition *in vivo* (195). A more recent study designed polyoxazoline block copolymer micelles-formulated vismodegib (POx-vismo), which actively functioned within the vascular compartment, thus displaying improved brain and tumor drug delivery and prolonged survival in medulloblastoma-bearing mice. This study has demonstrated the value of developing nanoparticle formulation to enhance anti-tumor efficiency, especially in brain tumor therapy (196). Indeed, Tylawsky’s group reported a fucoidan-based nanocarrier targeting endothelial tumor vasculature to promote blood-brain barrier crossing(197). This NPs encapsulating GDC-0449 significantly enhanced anti-tumor efficacy and drug exposure to healthy brain tissue with reduced bone toxicity. Given the important role of Hh signaling pathway across multiple cancers and TME, it is valuable to completely probe this strategy in many other Hh-dependent tumors.

### 5.3 Hedgehog antagonists in tumor immunotherapy

New insights into Hh signaling pathway regulation in TME may provide a novel opportunity for cancer immunotherapy. A clinical retrospective study showed that Hh targeting therapy was accomplished with increased infiltration of cytotoxic T cells in BCC (132). *In vitro* and *in vivo*, pharmacological inhibition of Hh pathway ameliorates several aspects of immunosuppressive TME *via* regulating the TAM polarization, CAF fate, T cell functions, and coordinating cell metabolic responses, suggesting that inhibition of Hh axis may serve as a promising strategy for immunotherapy.

In recent years, the application of PD-1/PD-L1 immune checkpoint inhibitors (ICIs) has revolutionized tumor therapy (133). However, most patients exhibit resistance to single-agent ICIs. Since GLI can both directly and indirectly regulate the expression of PD-L1 in tumor and immune cells, it is worthwhile to further interrogate its rational combination use with ICIs for tumor immunotherapy. Analysis of four independent clinical cohorts demonstrated that Hh signaling appears to be a negative biomarker for patients treated with ICIs (129). In an orthotopic PDAC mouse model, nanodrug encapsulated cyclopamine and chemotherapy drug paclitaxel significantly reversed primary resistance to ICIs and enhanced antitumor efficacy and prolonged animal survival, which at least partially due to Hh inhibition-triggered increased CD8^+^ T cells in TME (198). In hepatocellular carcinoma, Hh pathway inhibition with vismodegib or genetic depletion results in immunologic changes, including reduced PD-L1 expression, improved cytotoxic function of T cells and improved ICIs therapeutic efficiency (94, 96). In ovarian cancer, single therapy with anti-PD-L1 received modest effect, whereas combination with SMO inhibitor IPI-926, regained responses to immunotherapy, furtherly demonstrating the superiority in combination treatment (199). Importantly, their clinical combinations appear to be feasible, since two clinical trials observed encouraging responses to anti-PD-1 therapy, using pembrolizumab or cemiplimab, respectively, in patients with advanced basal cell carcinoma after treatment with Hh targeting inhibition, proving a proof-of-principle basis for rational combinations with ICIs in Hh abnormal tumors (200, 201). However, another clinical trial with patients suffering from metastatic or unresectable basal cell skin carcinoma demonstrated that pembrolizumab in combination with vismodegib treatment did not display increased responses, although it showed superior one-year progression-free survival compared with single pembrolizumab treatment (202). Thus, whether Hh inhibitors in combination with ICIs function as a more favorable therapeutical strategy in tumors and what specific type of tumors deserve further investigation.

## 6 Conclusions and perspectives

Aberrant Hh signaling pathway is implicated in a wide range of tumors. Emerging studies have described the associations between Hh signaling pathway and tumor immunity. These findings underscore that Hh signaling not only play an intrinsic role in tumor cells but also has an immunomodulatory role in TME by modulating the adaptions of macrophage polarity and maturation, T cell response, CAF function, and PD-L1 expression. In particular, the tumor-suppressive effects of Hh signaling in CAFs have recently been identified in a variety of tumors. Hence, Hh signaling activity must be finely balanced. As many compounds targeting Hh pathway are being developed in preclinical and clinical research for cancer therapy, it is valuable to detect changes in TME while monitoring the changes in tumor size and progression, which will not only be helpful to optimize treatment strategies, but also avoid drug-induced immunosuppression.

The presence of reciprocal regulatory crosstalk between cancer cells and TME, modulated by Hh pathway argues strongly for the development of rational combination strategy targeting Hh pathway and immune suppression, such as immune checkpoint inhibitors. However, our knowledge of the mutual determinism of Hh signaling and TME across diverse cancers remains largely limited. A better understanding detailed mechanisms in each tumor will allow to develop optimal combination therapy to improve clinical responses and decrease drug resistance. With the development of medicine and innovative technologies, such as single cell sequences and metabolomics, we are confident that targeting Hh pathway will result in more positive therapeutic outcomes.

NPs have emerged as a potential tool for anti-tumor therapies due to their prolonged retention time, reduced toxicity, and tissue targeted delivery (203). Recently, nanoparticle formulation of Hh-inhibitors has also been reported to target various aspects of tumor TME, such as CAFs and abnormal angiogenesis. Despite those significant advances, however, most of these NPs are still at the early stage and have not been evaluated in clinic. Besides, further studies are needed to focus on a comprehensive understanding of TME cell-specific effects of NPs-based Hh inhibition and identifying inhibitory efficiency in different Hh-dependent tumors. Improved understanding of Hh signaling in TME would pave the way for innovative NPs-based approaches in tumor therapy.

## Author contributions

JW conceived and designed the concept of this review article. XD and TH amended the manuscript. JW, BC and XL wrote the manuscript. All of the authors have contributed to the article and approved the submitted version.
